# Prenatal diagnosis of a *de novo* interstitial deletion of 11q (11q22.3 → q23.3) associated with abnormal ultrasound findings by array comparative genomic hybridization

**DOI:** 10.1186/s13039-014-0062-y

**Published:** 2014-09-25

**Authors:** Nian Liu, Jiong Yan, Xinlin Chen, Jieping Song, Bo Wang, Yanyi Yao

**Affiliations:** Prenatal Diagnosis Center, Hubei Maternal and Child Health Hospital, Wuhan, 430070 China

**Keywords:** Chromosome 11q deletion, Array CGH, G-band karyotyping, Fragile sites, *FRA11B*, *FRA11G*

## Abstract

**Background:**

Conventional G-band karyotyping offers low-resolution detection of chromosome abnormalities and cannot provide information about the involved genomic content. On the other hand, array comparative genomic hybridization can offer a rapid and comprehensive detection of genomewide gains and losses with higher resolution, thus providing the genetic basis for prenatal diagnosis of fetal abnormalities.

**Case presentation:**

A 35-year-old primigravid underwent cordocentesis at 28 weeks gestation due to the presence of polyhydramnios, intrauterine growth retardation, persistent right umbilical vein and mild stenosis of aortic arch at the ultrasound scan. Conventional G-band chromosome analysis revealed an apparently normal karyotype whereas the array CGH detected a *de novo* 8.97 Mb deletion at chromosome 11q22.3 → q23.3 and offered a precise characterization of the genetic defect.

**Conclusions:**

The array CGH detected a *de novo* interstitial 11q deletion with its precise location and size which could be missed or confused by G-band chromosome analysis. The breakpoint was close to the folate sensitive rare fragile site *FRA11B* and the aphidicolin inducible common fragile site *FRA11G*, the co-localization fragile site could have caused instability and constitutional chromosomal breakage. This case study indicates that array CGH is a useful technique for detecting small unbalanced chromosomal abnormalities and should be an integral part of prenatal diagnosis for fetal malformations.

## Background

The incidence of major structural birth defects in China is approximately 5.6% [1] and is associated with inherited or de novo genetic rearrangements and mutations as well as with maternal risk factors, such as advanced age, exposure to teratogenic factors, illnesses and infections. The detailed second trimester ultrasound scan can detect major fetal malformations and is offered for routine prenatal care. The genetic basis of abnormal ultrasound findings is important for prenatal diagnosis and counseling.

Conventional Giemsa-band (G-band) karyotyping on metaphase cells, which is the standard procedures used for prenatal cytogenetic diagnosis for over 40 years, can detect aneuploidy, unbalanced and apparently balanced structural rearrangements, and deletions/duplications of at least 5-10 Mb. The culturing process usually takes several days to a few weeks in order to generate the number of metaphase chromosomes enough for a full karyotype report. Moreover, G-band karyotyping lacks the resolution to assess the involved genomic content. These limitations hinder the inference of karyotype–phenotype predictions and the identification of candidate genes associated with fetal anomalies.

As in the majority of cases with ultrasound abnormalities the karyotype in the fetus is normal, thus demonstrating the need for additional diagnostic techniques with higher diagnostic capacity [2]. Array comparative genomic hybridization (array CGH) has been introduced in prenatal diagnosis to rapidly detect genomewide gains and losses with higher resolution [3]. It is a high throughput method which can detect copy number changes to a resolution of even as low as 1 Kb. Array CGH is rapidly replacing conventional G-band karyotyping in postnatal diagnosis of children with developmental, intellectual, and physical disabilities [4-7], but its application in prenatal diagnosis is still limited. Several groups have demonstrated that by applying array CGH for prenatal diagnosis of fetal ultrasound abnormalities, there was an increased detection rate over G-band karyotyping or other molecular cytogenetic techniques [8-11].

In this paper, we demonstrate the application of array CGH in prenatal diagnosis, which permits the rapid identification of a *de novo* interstitial deletion of 11q (11q22.3 → q23.3) in a fetus with abnormal ultrasound findings.

## Case presentation

A 35-year-old primigravid was referred to our hospital at 18 weeks of gestation for genetic counseling. The parents were healthy and nonconsanguineous. There was no family history of congenital malformations or genetic disorders. The mother was tested negative for toxoplasma, CMV, herpes, and rubella. No drugs or infections were reported during the course of the pregnancy. After informed about the possible risk of a chromosomal abnormality in the presence of advanced maternal age, the couple decided to receive noninvasive chromosomal aneuploidy screening. We performed sequencing analysis of the cell free DNA extracted from the maternal peripheral blood, and the result turned out to be negative for trisomy 13, 18 and 21.

**Figure 1:**
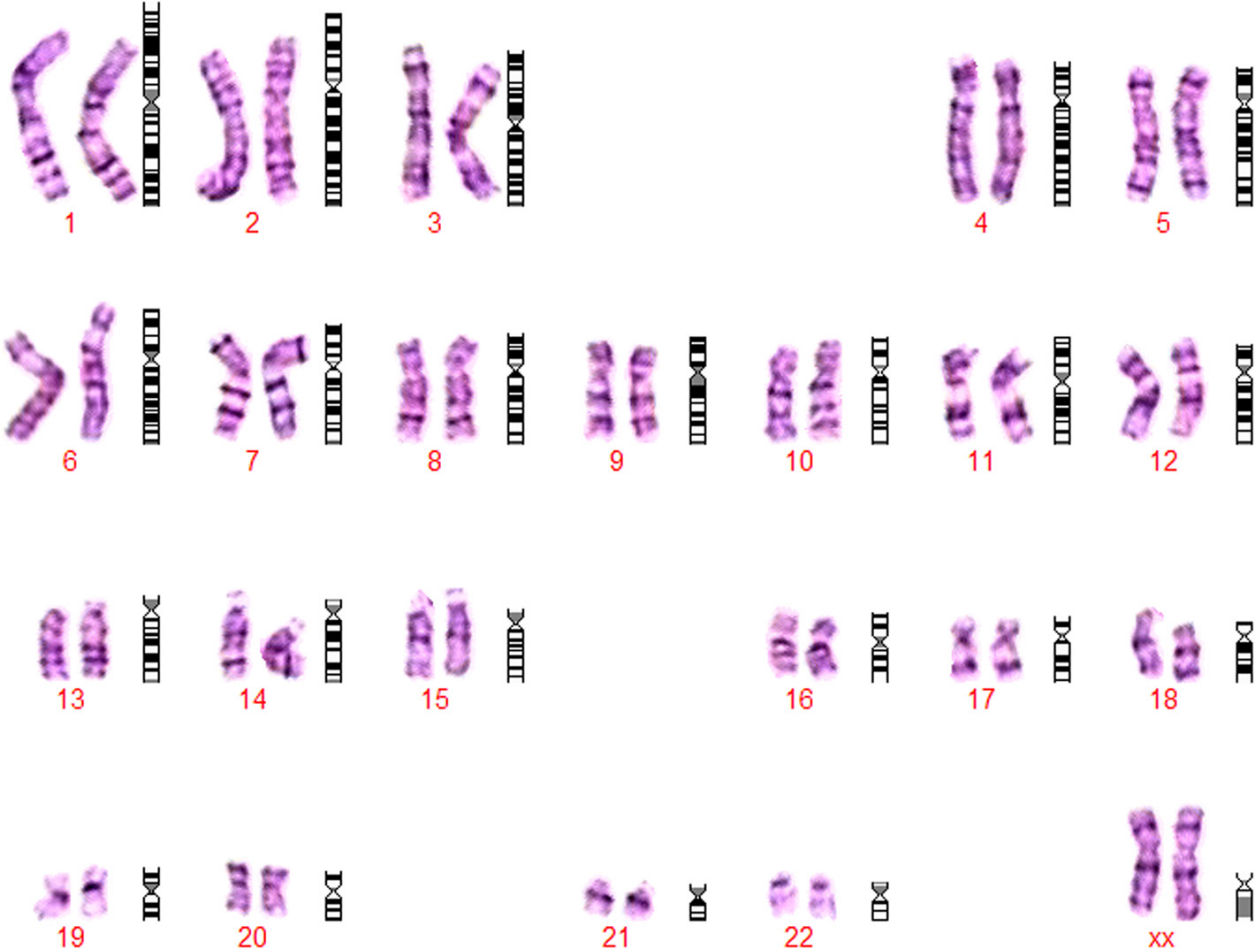
**G-banded karyotype of the fetus indicated an apparently normal 46,XX.**

**Figure 2:**
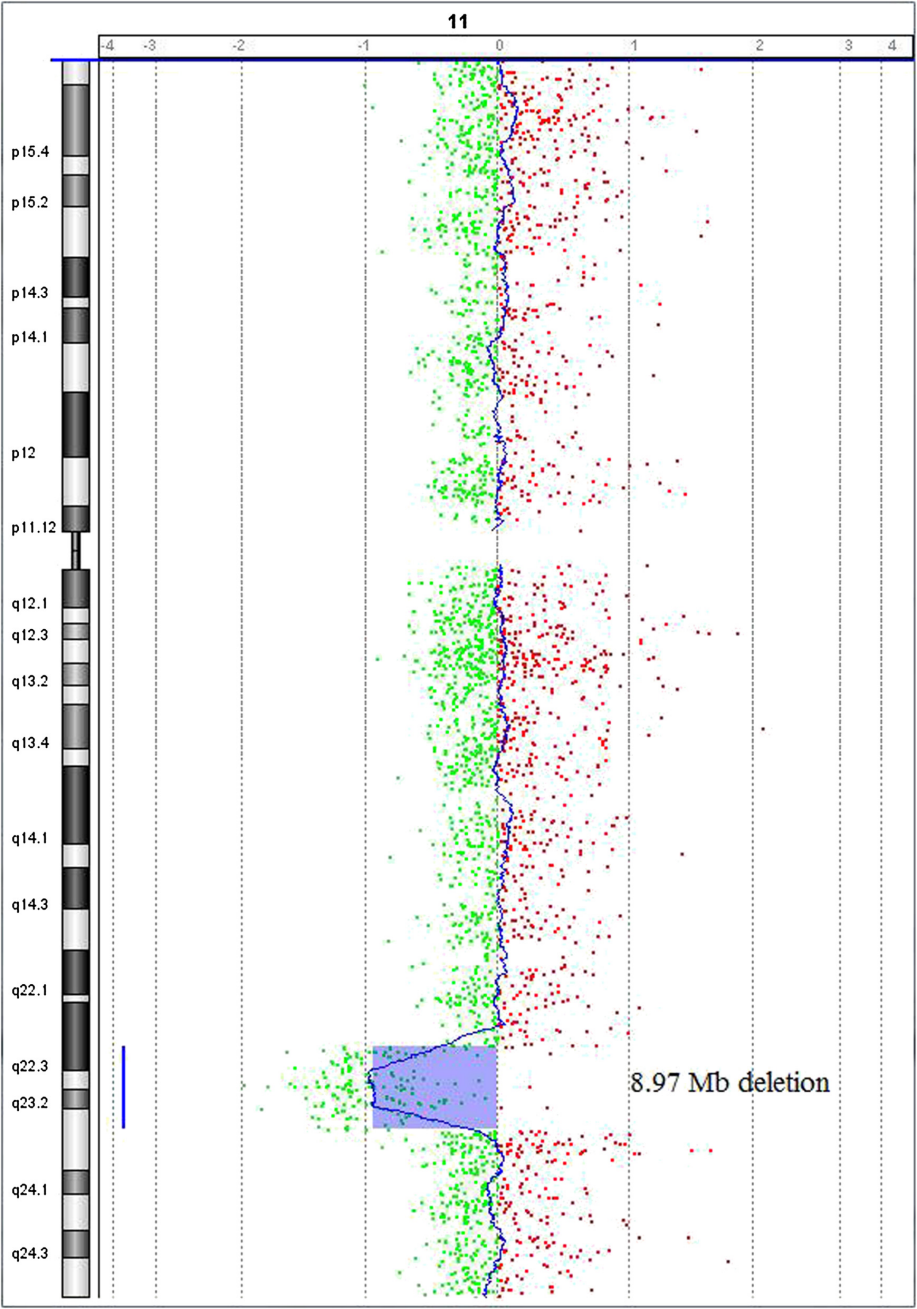
**Array CGH analysis of the fetus revealed a 8.97 Mb deletion at chromosome 11q22.3-11q23.3 or arr 11q22.3q23.3 (107,686,511-116,660,613)x1 (hg19; NCBI build 37).**

At 28 weeks of gestation the couple consulted our hospital again due to the presence of polyhydramnios, intrauterine growth retardation, persistent right umbilical vein and mild stenosis of aortic arch at the ultrasound scan. Interventional prenatal diagnosis was referred to the couple. A cordocentesis was carried out for prenatal diagnosis. Chromosome analysis was performed on cultured cord blood lymphocytes by Giemsa banding at approximately 350 band resolution. The cytogenetics revealed an apparently normal karyotype 46,XX (Figure 1) with the limited banding resolution. Array CGH using Agilent’s 8 × 60 K commercial arrays (Agilent Technologies, Santa Clara, CA, USA) was performed on DNA extracted from uncultured cord blood and a 8.97 Mb deletion was detected at chromosome 11q22.3-11q23.3 or arr 11q22.3q23.3(107,686,511-116,660,613)x1 (Figure 2). The molecular karyotyping refer to the Human Genome February 2009 (versions GRCh37, hg19) assembly. The array CGH analysis of the parental blood did not reveal any deletion at chromosome 11q, no balanced chromosomal rearrangements or inversions were found by G-band karyotyping.

After genetic counseling, termination of pregnancy was performed at parent’s request at 30 weeks of gestation. A female fetus was delivered with no apparent phenotypic abnormalities. Autopsy was rejected by the parents.

## Conclusion

The majority of chromosome 11 long arm deletions are at the terminal region, which is associated with a clinically well-described phenotype, called Jacobsen syndrome. Whereas the interstitial deletions of chromosome 11q are rarely observed, it may being resulted from a direct *de novo* deletion or familial inheritance [12-14]. To our knowledge, only 33 cases with interstitial 11q deletion have been reported in literature and previous reports of such cases have been associated with a wide variability of phenotypic findings. Most of these cases had not been analyzed with a molecular method, thus the phenotype-genotype correlation was not very clear.

Among the 33 cases previously described, only six were characterized with a molecular definition (Table 1). Danijela Krgovic reported a deletion of chromosome 11q22.3 in a 5-year-old girl with mild mental retardation, developmental and speech delay, facial dysmorphism, ptosis, hypertelorism, low-set dysplastic ears, prominent forehead and hypoplastic corpus callosum [15]. Peining Li reported a deletion of chromosome 11q14.1 → q23.2 involving the *FZD4* gene in a patient with growth retardation, facial anomalies, exudative vitreoretinopathy (EVR), cleft palate, and minor digital anomalies [16]. Daniela Melis reported a deletion of chromosome 11q13.5 → q14.2 in a 5-year-old boy with low frontal hairline, flat profile, round face, full cheeks, periorbital fullness, hypertelorism, broad nasal bridge, down-turned corners of the mouth and developmental delay [17]. Rebecca L. Sparkes reported a maternally inherited 11q14.3 → q22.3 deletion of a male fetus, the ultrasound scan revealed choroid plexus cysts, echogenic intracardiac foci, mild polyhydramnios, a relatively enlarged right atrium with abnormal cardiac axis, small cerebellum and left talipes equinovarus. The 38-year-old mother with a 17, 3 Mb deletion (from nt 89,492,818 to nt 106,832,040) and a 0.9 Mb duplication (from nt 88,258,744 to nt 89,103,489) in 11q21 → q23 was of normal intellect and healthy only with a surgically repaired bilateral club foot and high myopia [14]. Josephine Wincent reported a deletion of chromosome 11q13.4 → q14.3 in a boy with microcephaly, ptosis and developmental delay [18]. Renata Nacinovich followed up a boy with 11q14.3 → q22.3 deletion from early infancy to adolescence, the proband was born with hypotonia and minor dysmorphisms (submucous cleft palate), his height growth and cognitive development were at the lower limit during childhood [19].

**Table 1:** **Clinical manifestations related to interstitial 11q deletions (molecularly defined cases)**

**Segment deleted**	**Clinical manifestations**
11q22.3	mild mental retardation, developmental and speech delay, facial dysmorphism, ptosis, hypertelorism, low-set dysplastic ears, prominent forehead, hypoplastic corpus callosum
11q14.1 → q23.2	growth retardation, facial anomalies, exudative vitreoretinopathy (EVR), cleft palate, minor digital anomalies
11q13.5 → q14.2	low frontal hairline, flat profile, round face, full cheeks, periorbital fullness, hypertelorism, broad nasal bridge, down-turned corners of the mouth, developmental delay
11q14.3 → q22.3	choroid plexus cysts, echogenic intracardiac foci, mild polyhydramnios, a relatively enlarged right atrium with abnormal cardiac axis, small cerebellum, left talipes equinovarus (Prenatal ultrasound result)
11q13.4 → q14.3	microcephaly, ptosis, developmental delay
11q14.3 → q22.3	mild developmental delay, submucous cleft palate

As we mentioned above, due to the heterogeneity in size and position of the deletions, it is not easy to define a distinctive genotype/phenotype of the interstitial 11q deletion. Most of the patients with an overlapping deletion of this region had mild to severe mental retardation and developmental delay, usually depending on the size and position of the deletion. However, because our case was diagnosed in uterus, it was not possible to investigate the mental and developmental delay. In the only prenatal diagnosed report, mild polyhydramnios and a suspected structural cardiac malformation was described [14], our case also shows the similar ultrasound results.

In the deleted region of this case, about 30 genes with already known or unknown functions are mapped [http://www.ncbi.nlm.nih.gov/]. In a proportion of patients with Jacobsen syndrome (terminal 11q deletions) the breakpoints cluster in chromosomal subband 11q23.3 [20-22], a breakage-prone region which encompasses both the folate sensitive rare fragile site FRA11B and the aphidicolin inducible common fragile site *FRA11G* [20,23-25]. In our case, the deleted region maps at 11q22.3 → q23.3, the breakpoint is on the proximal side of *FRA11G* and *FRA11B*, indicating that the co-localization fragile site could have caused instability and constitutional chromosomal rearrangements in vivo [23,26]. Aphidicolin inducible common fragile sites do not break at defined sequences but in breakage-prone regions up to 10 Mb where the break is most likely to appear [25,27,28]. The molecular basis of common fragile site fragility has not been fully clarified. Common fragile sites contain more areas of high DNA torsional flexibility with more highly AT-dinucleotide-rich islands than neighbouring non-fragile regions. The inconsistency in DNA replication progression between fragile and flanking non-fragile regions might contribute to occurrence of breaks at these fragile sites.

Folate sensitive fragile sites are caused by expansive mutations of the normally occurring CCG/CGG trinucleotide repeat sequences adjacent to a CpG island [29,30]. In majority of normal individuals this CCG/CGG repeat is present in 8-80 copies [23]. The repeat can expand to 85–100 copies as a premutation, without cytogenetic expression of the fragile site. During the premutation phase, the repeats become highly unstable when transmitted to the next generation. The offspring may then have a longer or a shorter extension of the repeat sequence than the parent [31]. In individuals with cytogenetic expression of the *FRA11B* the repeat is expanded to several hundred copies. This expansion is unstable and dependent upon the length of the repeat tract: the longer the tract, the higher the instability and probability of further expansion. It is hypothesized that hypermethylation of the expanded CCG/CGG trinucleotide on chromosome 11 could delay DNA replication of this fragile site, resulting in a break and/or impaired DNA replication [32].

The chromosome band 11q23 is often involved in multiple tumor associated rearrangements. On the distal side of FRA11B lies the *MLL* gene. The *MLL* gene regulates the *HOX* gene expression by directly binding to the promoter sequences. Translocations involving the *MLL* gene have been found in acute myeloid leukemia (AML), acute lymphoblastic leukemia, or mixed linkage leukemia (MLL) [33]. The region could be considered as a hotspot of various tumors and chromosomal recombination or breakage.

We report the prenatal diagnosis of a de novo interstitial deletion of 11q (11q22.3 → q23.3) performed by array CGH in a fetus with polyhydramnios, intrauterine growth retardation, persistent right umbilical vein and minor cardiac abnormalities at 28 weeks of gestation. In this study, we used combinations of classic G-band karotyping, with array CGH methods to undertake a genome-wide screening for chromosome aberrations. The array CGH successfully detected the 8.9 Mb deletion with its precise location which could be missed or confused by G-band chromosome analysis. It provides valuable information for genetic counselors to achieve molecular diagnosis of prenatal anomalies and to make more accurate predictions about the clinical outcomes. The results indicate that array CGH is a useful technique for detecting small unbalanced chromosomal abnormalities and should be an integral part of prenatal diagnosis for fetal malformations.

## Consent

Written informed consent was obtained from the parents of the proband for publication of this Case Report and any accompanying images. The consent form was approved by the ethical committee of Hubei Maternal and Child Health Hospital, China. A copy of the written consent is available for review by the editor of this journal.
